# MOOCs (massive online open courses) as innovative tools in education in infection prevention and control: reflections from the first MOOC on Ebola

**DOI:** 10.1186/2047-2994-4-S1-O16

**Published:** 2015-06-16

**Authors:** R Ruiz De Castaneda, A Valticos, D Pittet, A Flahault

**Affiliations:** 1 University of Geneva, Faculty of Medicine, Geneva, Switzerland; 2 University of Geneva, Faculty of Medicine, Institut of Global Health, Geneva, Switzerland; 3 University of Geneva and University Hospitals of Geneva, Department of Prevention and Infection Control, Geneva, Switzerland

## Introduction

MOOCs and their platforms are revolutionising education at a global scale. Courses from world-class universities are now accessible online for all for free. Thousands of learners can interact among them and with experts via the forums used in MOOCs. MOOCs are a source of *Big data* to explore performances of learners. The number of MOOCs on health and life sciences is increasing targeting the general public, health professionals and/or decision makers. Continuing education of health professionals improves the quality of healthcare and MOOCs could play a role where access to quality education is limited.

## Objectives

Improve the understanding of Ebola by the general public, but especially by health professionals and decision makers.

## Methods

The UNIGE and UNF3S in collaboration with experts from different disciplines and academic or political institutions, international organisations and NGOs (37 experts from 19 institutions), have produced the first MOOC on Ebola. It was set-up on the platform *FUN* at the end of 2014 with a transdisciplinary program of five weeks with 57 video-lectures. A second run on *Coursera* will start imminently.

## Results

1988 enrolments from 66 countries. 330 of these took the final evaluation and 260 passed it. France and Switzerland led the number of participants with a total of 588. 526 participants came from Africa, 332 from West Africa and 120 from Central Africa. Guinea had 79 participants. 332 participants provided no data on their origin. The majority of participants were in their thirties and had a master (625) or a doctorate (486). More than 85% out of 95 participants who completed the final survey, rated the course as excellent or very good.

## Conclusion

These are the first insights on the potential of MOOCs as innovative tools in health crisis. The number of enrolments may be limited by this new French platform. The second run of the MOOC on *Coursera* will extend these results. Although this MOOC addressed some aspects of prevention and infection control, specific MOOCs in this field are needed to train a larger number of health professionals and evaluate them to ensure that specific practices are correctly implemented where they are most needed.

## Disclosure of interest

None declared.

